# Synthesis of a multifunctional hard monomer from rosin: the relationship of allyl structure in maleopimarate and UV-curing property

**DOI:** 10.1038/s41598-018-20695-5

**Published:** 2018-02-05

**Authors:** Yanju Lu, Zhendong Zhao, Liangwu Bi, Yuxiang Chen, Jing Wang, Shichao Xu

**Affiliations:** 1 Institute of Chemical Industry of Forest Products, CAF, National Engineering Laboratory for Biomass Chemical Utilization, Key and Open Laboratory on Forest Chemical Engineering, SFA, Key Laboratory of Biomass Energy and Material, Jiangsu Province Nanjing, 210042 China; 2 Research Institute of Forestry New Technology, CAF, Beijing, 100091 China

## Abstract

Rosin is an important forestry resource with a specific three-membered phenanthrene ring structure, which can improve the mechanical resistance of polymer coatings. In this paper, a high purity rosin monomer, tri-allyl maleopimarate containing three allyl groups has been synthesized. The yield of the monomer product was 93.2% with the purity of 96.1%. The structure of the synthesized monomer was characterized through gas chromatography (GC), mass spectrometry (MS), hydrogen nuclear magnetic resonance spectroscopy (^1^H NMR), carbon nuclear magnetic resonance spectroscopy (^13^C NMR) and elemental analysis. Additionally, we present new experimental results regarding the polymerization reaction under ultraviolet (UV) irradiation. The cured film of tri-allyl maleopimarate exhibited good mechanical properties. The films were also characterized through thermogravimetric (TG) and differential scanning calorimetry (DSC) analyses and a mechanism for polymerization was proposed. Overall, a facile catalytic process for the valorization of rosin in the field of UV polymerization is reported.

## Introduction

The total production of rosin in China is about 800,000 t/a. The main components in rosin are resin acids, which make up about 90% (mass fraction), and neutral compounds, forming the remaining 10%. Resin acids can be used as coatings^1–4^, epoxy resins^5,6^ and adhesives^7^. Their specific three-membered phenanthrene ring structure confers to the polymers the characteristics of good rigidity, flexibility and corrosion resistance.

Abietic-type acids are the main components in rosin, accounting for 80%–90% of the total rosin acids^8^. A specific chemical property of abietic-type acids is their automatic molecular transformation under acid catalysis and heat, generating levopimaric acid with a yield close to 100%. In the molecular structure of levopimaric acid, conjugated double bonds can be formed, allowing functional groups to graft through Diels–Alder reactions^9^, such as the Malay reaction.

Maleic rosin is the product of the Malay reaction carried out using abietic-type acid resin and maleic anhydride as raw materials. The addition of Maleic rosin has been shown to improve the properties of rosin, including its softening point and stability. Malay rosin was first produced in 1930s and at present is used mainly as an intermediate, particularly in the fields of paper making, painting, printing ink, architecture, chemical engineering, and fruit surface waxing^10,11^. Several studies have been reported in literature concerning the Malay reaction of rosin. Ray *et al*.^12^ synthesized the rosin maleic anhydride adduct and then condensed it with hexamethylenediamine to obtain an amino acid. Kazakova *et al*.^13^ synthesized Malay rosin and used it to synthesize a new group of maleopimaric acid amides containing fragments of methyl ethers of amino acids, aliphatic amines, imidazole, and methylpiperazine. Bei and Yuvchenko^14^ synthesized maleopimaric N-(n-alkyl)imides using maleopimaric acid with primary aliphatic amines as starting materials. The reaction between the methyl ester of maleopimaric acid and dimethyldioxirane resulted in regioselective oxidation of the bridging double bond, forming the 13(15)-en-14 S -hydroxy derivative^15^. Meng *et al*.^16^ synthesized Malay rosin via a two-step method. First, resin acid was separated from rosin through salt formation with cyclohexy lamine. Then, using *p*-toluene sulfonic acid as catalyst, the resin acid reacted with maleic anhydride through the Diels–Alder reaction. Wang *et al*.^17^ synthesized Maleopimaric acid under microwave irradiation using gum rosin of *Pinus massoniana* and maleic anhydride as starting materials. The effects of different conditions on the Diels–Alder addition were investigated.

One of its important high added-value applications is the production of highly reactive monomers for polymerization. For example, Lewis *et al*.^18^ synthesized maleic pimaric acid with one vinyl double bond. Atta *et al*.^19^ synthesized the monomer of maleic pimaric acid with two vinyl double bonds. Wang *et al*.^20^ synthesized the monomer of allyl maleopimarate, and characterized the structure and properties of the product and the ultraviolet (UV) cured product. Yu *et al*.^21^ carried out the reaction between acrylic rosin with 2-hydroxyethyl methacrylate. The product possessed double bonds and was used as a cross-linking monomer for the preparation of polystyrene microspheres through suspension polymerization, replacing the traditional monomer, divinyl benzene.

It was found that the introduction of rosin had a strong influence on the physical properties of the monomers. Liu *et al*.^22^ studied the UV curing reaction of malay pimaric acid, and found that the large fused ring structure of malay pimaric acid could increase the thermal decomposition temperature. The UV curing reaction of maleic acid was also studied and it was found that the UV cured product had a lower thermal decomposition temperature but the flexibility was improved^23^.

In our previous work, we also demonstrated the improvement of film properties through the introduction of the rosin structure. Allyl resinate (AR), a high purity monomer, has one allyl group, and can be polymerized to generate a film with good properties^24^. Furthermore, another new rosin monomer (allyl acrylpimarate, AA) with two allyl groups was synthesized by our group. The surface drying time of the UV cured product of AA, which has two allyl groups, is shorter than the UV cured product of AR in the same conditions. We also found that relative speed, pencil hardness and thermal stability of the UV cured products could be further improved.

On the basis of our previous research, we believe that the introduction of different numbers of allyl groups in the resin structure leads to a completely different polymerization mechanism, thus generating polymer products with specific mechanical properties. As a result of the above considerations, in this study we tried to synthesize a highly reactive monomer with three outside double bonds. Such a material has not been previously reported in UV polymerization studies.

In this paper, a new methodology for the synthesis of tri-allyl maleopimarate monomer was investigated. The tri-allyl maleopimarate monomer has three reactive groups. The structures of the product and byproduct have been characterized by mass spectrometry (MS), nuclear magnetic resonance spectroscopy (NMR), etc. The physochemical properties of the UV cured product of tri-allyl maleopimarate were tested according to the Chinese standards. The reaction mechanism of the UV curing reaction by tri-allyl maleopimarate was also studied.

## Results and Discussion

### Synthesis of tri-allyl maleopimarate

Sodium maleopimarate and allyl chloride were used as raw materials to synthesize tri-allyl maleopimarate. Hexadecyl trimethyl ammonium bromide, was used as phase-transfer catalyst. In order to improve the yield of tri-allyl maleopimarate, different reaction conditions were studied, such as the microwave power, amount of phase transfer catalyst, amount of additives of allyl chloride, and reaction temperature. The effects of different parameters on the yield of the product are shown in Table 1.

**Table 1:** Influences of reaction parameters on the yield of tri-allyl maleopimarate by microwave heating.

Items	Reaction conditions	Yield (%)
Microwave power (W)	300	38.6
	400	59.1
	500	22.7
	600	15.9
Reaction temperature (°C)	40	43.2
	45	45.5
	50	47.7
	55	59.1
	60	56.8
Reaction time (h)	2	36.4
	2.5	43.2
	3	45.5
	3.5	47.7
	4	59.1
	4.5	57.3
*n*(allyl chloride)/*n*(sodium maleopimarate)	1.0	22.7
	1.5	34.1
	2.0	59.1
	2.5	81.9
	3.0	93.2
	3.5	61.4
Catalyst dosage (%)	2	45.5
	3	54.5
	4	63.6
	5	93.2
	6	79.5

Microwave power has a great influence on the yield and purity of tri-allyl maleopimarate. The optimal microwave power was 400 W, which corresponded to a yield of 59.1%. With regards to temperature, the reaction could not take place below 40 °C. For higher temperatures, the yield of tri-allyl maleopimarate increased with increasing reaction temperature. The yield of the product reached the maximum when the temperature up to 55 °C. A further increase in temperature caused a reduction in the yield due to the emergence of secondary reactions. The yield of tri-allyl maleopimarate at different reaction time was also investigated. The product yield increased to its maximum value of 59.1% at 4 h. Further increases in the reaction time caused a decrease in the yield. The molar ratio of raw materials was also tested. At lower ratios (≤1:1), product yield increased slightly. A maximum yield of 93.2% was observed at a ratio of 3:1. The yield of product decreased quite sharply at ratios >3:1. The yield of tri-allyl maleopimarate first increased then stabilized with increasing amounts of catalyst. The optimum catalyst amount was 5%, while the yield of the product was 93.2%. The optimum microwave power, reaction temperature, reaction time, *n*(allyl chloride)/*n*(sodium maleopimarate) and catalyst amount were 400 W, 55 °C, 4 h, 3:1 and 5%, respectively. The yield of tri-allyl maleopimarate was 93.2% under the optimum reaction conditions.

An interesting observation was made during the separation of tri-allyl maleopimarate from the reaction mixture. Following extraction with *n*-hexane, the monomer products were accompanied by a white precipitate byproduct. More detailed information concerning the byproduct, including its physiochemical and UV-curing properties, is provided in the the Supplementary Materials.

### Physicochemical properties of the synthesized tri-allyl maleopimarate

#### FTIR analysis of tri-allyl maleopimarate

**Figure 1:**
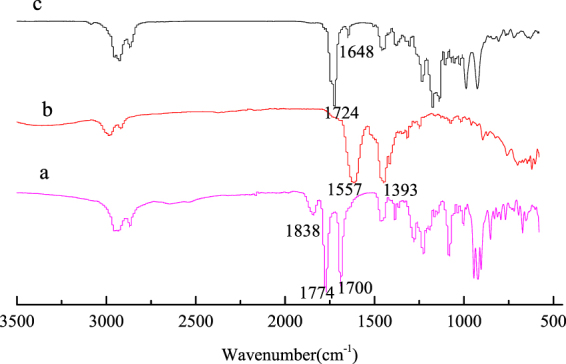
FTIR spectra of tri-allyl maleopimarate using sodium maleopimarate as raw material, (**a**) maleopimaric acid anhydride; (**b**) sodium maleopimarate; (**c**) tri-allyl maleopimarate.

The infrared spectra of material and products are shown in Fig. 1. Figure 1(a) represents maleopimaric acid anhydride. The peaks at 1838 cm^−1^ and 1774 cm^−1^ were the stretching coupling vibration of the C=O bond in acid anhydride. The peak at 1700 cm^−1^ represents stretching vibration of the C=O bond in C18. Figure 1(b) is the FTIR spectrum of sodium maleopimarate. It can be seen that the peaks relative to the stretching coupling vibration of acid anhydride at 1838 cm^−1^ and 1774 cm^−1^, and to carboxyl absorption, at 1700 cm^−1^, disappeared. The peaks at 1557 cm^−1^ and 1393 cm^−1^ are attributable to the symmetric and anti-symmetric stretching vibration peaks of the —CO^2−^ group. Figure 1(c) is the FTIR spectrum of tri-allyl maleopimarate. The absorption peak of carboxyl at 1700 cm^−1^ disappeared. Several new peaks appeared at 1724 cm^−1^, 1648 cm^−1^, and at 3080 cm^−1^, which are due to the adsorption of the ester, unsaturated C=C double bonds, and the =C—H group, respectively, indicating the successful introduction of the allyl group into the product. The byproduct was also analyzed and its FTIR spectrum is shown in Figure S1 of the Supplementary Materials.

#### Gas chromatographic analysis of tri-allyl maleopimarate

**Figure 2:**
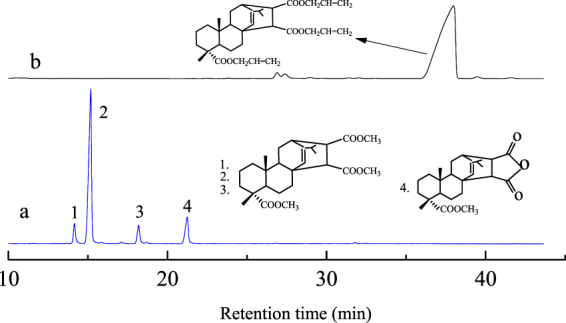
Gas chromatograms of tri-allyl maleopimarate using sodium maleopimarate as raw material, (**a**) maleopimaric acid anhydride; (**b**) tri-allyl maleopimarate.

The raw materials and products were identified by GC analysis, which shown in Fig. 2. Figure 2(a) shows the GC results of the maleopimaric acid anhydride. The peaks for each component are: peaks 1, 2 and 3 are all due to malay pimaric acid trimethyl, and peak 4 is due to malay pimaric acid methyl ester. Figure 2(b) is the GC trace of tri-allyl maleopimarate and shows that after the esterification reaction the only detectable peak is the one associated with the product. According to the GC measurements, the content of tri-allyl maleopimarate is 96.1%, measured at a retention time of 37.9 min, and the content of the allyl maleopimarate byproduct is 3.9%, measured at a retention time of 26.7 min. The GC spectrum of the purified byproduct is shown in Figure S2 of the Supplementary Materials. It can be seen that the raw material peak has completely disappeared and the purity of the product is high.

#### MS analysis of tri-allyl maleopimarate

**Figure 3:**
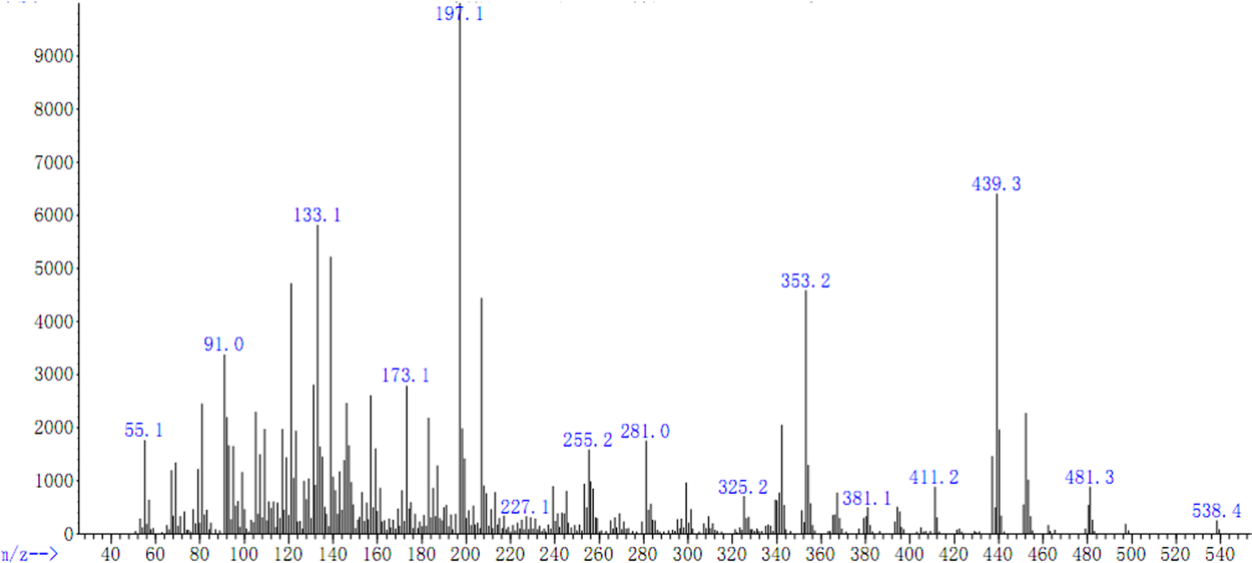
MS of tri-allyl maleopimarate.

**Figure 4:**
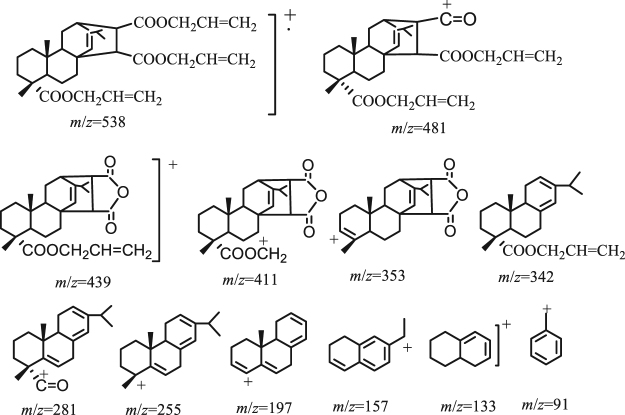
Possible fragment ions of tri-allyl maleopimarate.

Typical fragmentation patterns of tri-allyl maleopimarate are shown in Figs 3 and 4. The fragment ion with molecular weight 538 represents the molecular ion [M]^+^. Loss of an allyl group from the tri-allyl maleopimarate substituent resulted in the formation of the [M–OCH_2_CHCH_2_]^+^ fragment. Loss of two allyl groups yielded the fragment ion 439, which retains the complete structure of allyl maleopimarate. The formation of the fragment ion with molecular weight 342 is the result of the reverse Diels–Alder reaction. The base peak is the fragment ion 197. Other fragmentation patterns of allyl maleopimarate are shown in Figures S3 and S4 of the Supplementary Materials.

#### ^1^H NMR analysis of tri-allyl maleopimarate

**Figure 5:**
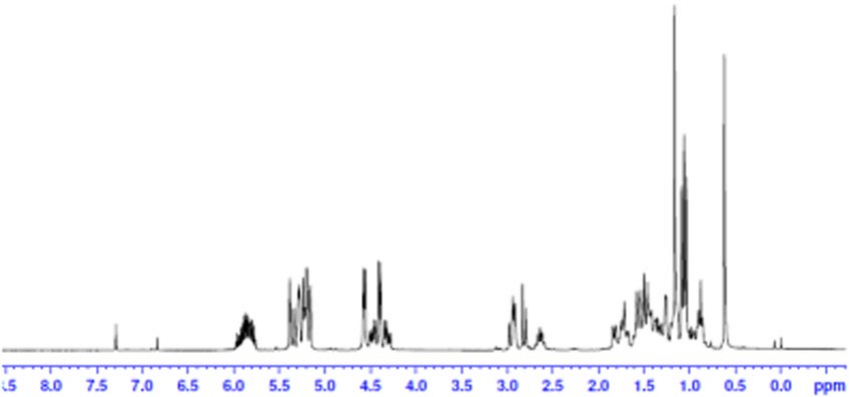
^1^H NMR spectrum of tri-allyl maleopimarate.

The chemical structures of tri-allyl maleopimarate (Fig. 5) and its byproduct (allyl maleopimarate) (Figure S5) were studied by ^1^H NMR. The significant signals of ^1^H NMR observed when analyzing tri-allyl maleopimarate can be summarized as follows: *δ* 5.88 (C26—H), *δ* 5.87 (C29—H) and *δ* 5.87 (C32—H); double peaks of each 2H at *δ* 4.41 (C25—H), *δ* 4.56 (C28—H) and *δ* 4.58 (C31—H), *δ* 5.29~5.16 (C27—H, C30—H, C33—H). The results also proved the successful formation of the three vinyl double bonds in the product. The other ^1^H NMR signals can be summarized as follows (*δ*_H_, ppm): *δ* 1.46 (m, 1H, C2—H), 1.26 (m, 1H, C2—H), 1.71 (t, 1H, C3—H), 1.58 (1H, C3—H), 2.83 (1H, C5—H), 1.43 (1H, C1—H), 1.25 (1H, C1—H), 1.54 (1H, C6—H), 1.25 (1H, C6—H), 1.49 (1H, C7—H), 1.25 (1H, C7—H), 1.42 (1H, C9—H), 5.38 (1H, C14—H), 2.79 (1H, C12—H), 1.54 (1H, C11—H), 1.25 (1H, C11—H), 5.34 (m,1H, C15—H), 1.06 and 1.03 (d, 3H, C16—H), 1.08 and 1.06 (d, 3H, C17—H), 0.62 (s, 3H, C19—H), 1.16 (s, 3H, C20—H), 2.92 (d, 1H, C21—H), 2.94 (s, 1H, C22—H), 4.41 (d, 2H, C25—H), 5.88 (m, 1H, C26—H), 4.56 (d, 2H, C28—H), 5.87 (m, 1H, C29—H), 4.58 (d, 2H, C31—H), 5.85 (m, 1H, C32—H), 5.29~5.16 (d, 6H, C27—H, C30—H, C33—H).

#### ^13^C NMR analysis of tri-allyl maleopimarate

**Figure 6:**
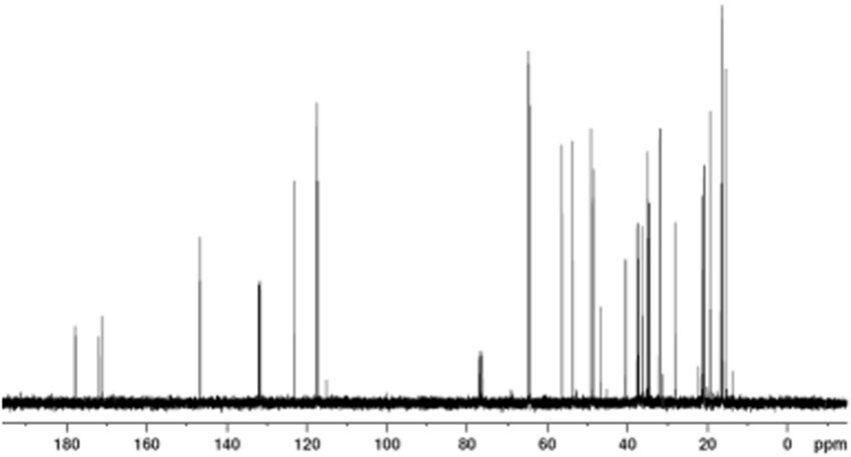
^13^C NMR spectrum of tri-allyl maleopimarate.

The ^13^C chemical shifts of tri-allyl maleopimarate were measured (Fig. 6 with chloroform; used as internal reference). In the ^13^C NMR spectrum, the signals of the C=O atoms, C-18, C-23 and C-24, appear at *δ* = 177.9, 171.2, and 171.9 ppm, respectively. The signals of the allyl double bonds appear at *δ* = 131.9 (C-26), 131.7 (C—29), 131.7 (C—32), 117.3 (C—27), 117.5 (C—30), 117.6 ppm (C—33). The ^13^C NMR chemical shifts and the ^13^C NMR signals relative to the byproduct (allyl maleopimarate) formed from tri-allyl maleopimarate are shown in Figure S6 and Table S1 of the Supplementary Materials.

The ^13^C NMR signals of tri-allyl maleopimarate are reported in Table 2.

**Table 2:** ^13^C NMR chemical shift of each carbon atom from tri-allyl maleopimarate.

Carbon number	*δ*	Carbon number	*δ*
1	37.5	18	177.9
2	16.5	19	20.1
3	37.2	20	20.8
4	40.3	21	48.4
5	53.7	22	56.3
6	19.2	23	171.2
7	36.3	24	171.9
8	35.1	25	64.3
9	49.0	26	131.9
10	46.6	27	117.3
11	28.0	28	64.6
12	34.9	29	131.7
13	146.6	30	117.5
14	34.5	31	64.6
15	123.2	32	131.7
16	21.3	33	117.6
17	22.1		

#### Elemental analysis

The results of the elemental analysis, expressed in percentages, of tri-allyl maleopimarate are as follows: *ω*(C) = 72.7(73.6), *ω*(H) = 8.6(8.6), *ω*(O) = 18.3(17.8), where the values in parentheses represent the theoretical data. A good agreement between the analysis results and the theoretical data can be noticed.

#### Physical properties of tri-allyl maleopimarate

Table 3 shows the physiochemical properties of tri-allyl maleopimarate. The product was a viscous liquid with a viscosity of 8.5 × 10^3^ mPa·s at 25 °C. Its density and acid value were 1.1097 × 10^3^ kg/m^3^ and 2.5 mg/g, respectively.

**Table 3:** Physical properties of tri-allyl maleopimarate.

Items	Experimental data
Appearance	White
Characters	Liquid
Viscosity (mPa·s, 25 °C)	8.5 × 10^3^
Density (kg/m^3^, 25 °C)	1.1097 × 10^3^
Acid value (mg/g)	2.5

#### UV-curing performance of tri-allyl maleopimarate

The UV-curing reaction conditions were as follows: illumination distance 4.5 cm, illumination intensity 100%, photoinitiator 6512.

#### FTIR monitoring of tri-allyl maleopimarate conversion

**Figure 7:**
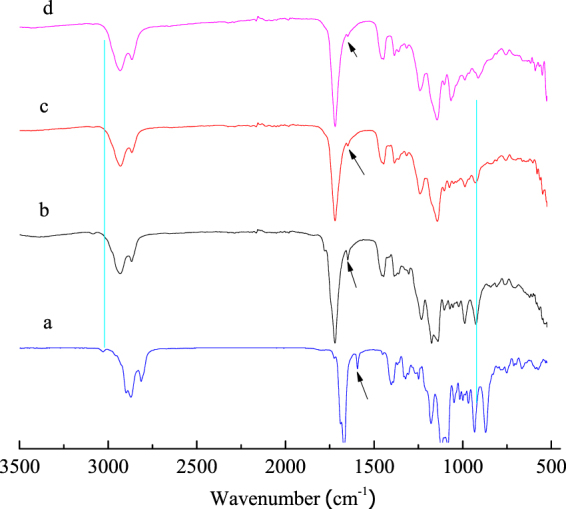
FTIR spectra of tri-allyl maleopimarate at different curing time, (**a**) 0 min; (**b**) 10 min; (**c**) 20 min; (**d**) 30 min.

The FTIR spectra of the product of tri-allyl maleopimarate polymerization under UV irradiation for different curing times are shown in Fig. 7. The C=C double bonds were gradually polymerized with the increase of curing time. The areas of the corresponding peaks at 1648 cm^−1^ and 3080 cm^−1^ decreased accordingly. It was also found that the area of the peak relative to the carbonyl group (1720 cm^−1^) remained the same after UV irradiation; however, it shifted towards a higher wavenumber because the disappearance of the C=C double bond cause the destruction of the its conjugate. As the curing reaction proceeds the C=C double bond becomes a C-C single bond, therefore, the characteristic absorption peak of =C-H at 3080 cm^−1^ gradually disappeared. Nonetheless, the absorption peak relative to the C=C double bond cannot disappear completely, regardless of the extent of the UV curing time, because the steric hindrance resulting from cross-linking polymerization prevents the reaction of all the double bonds. The FTIR spectra of the product polymerized from allyl maleopimarate (byproduct) before and after curing under UV irradiation are shown in Figure S7; the specific parameters were also reported in the Supplementary Materials.

#### Effects of curing conditions on the surface drying time from tri-allyl maleopimarate

Table 4 shows the surface drying time of tri-allyl maleopimarate with increasing dosages of photo initiators. The reaction conditions were as follows: illumination distance 4.5 cm, illumination intensity 100%, photo initiator 6512. The surface drying time of tri-allyl maleopimarate decreased rapidly with increasing photoinitiator dosage when the dosage was lower than 4%. That is because the number of primary free radicals, which can initiate chain growth and termination, increases with increasing photoinitiator dosage after irradiation with UV light. Further increases of the photoinitiator dosage, from 5% to 10%, did not affect the drying time. For photoinitiator dosages ≤5%, the surface drying time of allyl maleopimarate was obviously shorter than that of tri-allyl maleopimarate. This is because tri-allyl maleopimarate had undergone cross-linking polymerization and generated a space grid structure; by contrast, the byproduct of allyl maleopimarate has only one reactive vinyl group, which leads to a lower drying time (see Table S2 of the Supplementary Materials). Additionally, the conversion of the anhydride to carboxylic acid during photo polymerization is expected to introduce interchain hydrogen bonding interactions and further reduce the drying time.

**Table 4:** Influence of different curing conditions on surface drying time from tri-allyl maleopimarate.

Curing conditions		Surface drying time(s)
Photo-initiator dosage (%)	1	200
	2	160
	3	100
	4	25
	5	15
	6	8
	7	6
	8	6
	9	6
	10	5
Illumination distance (cm)	4.5	15
	9.0	35
	13.5	55
	18.0	75
	22.5	95
Illumination intensity (%)	50	135
	60	75
	70	40
	80	30
	90	20
	100	15

From Table 4 it can be seen that the surface drying time increased linearly with the illumination distance, because as the distance increased the illumination intensity, and consequently the number of primary free radicals generated, increased.

Table 4 shows the influence of the illumination intensity on the surface drying time of tri-allyl maleopimarate. Surface drying time decreased rapidly with increasing illumination intensity because, when all other conditions were kept the same, the number of photons received per unit area increased with increasing illumination intensity and more primary free radicals were generated, thus decreasing the surface drying time.

#### Thermal stability evaluation

**Figure 8:**
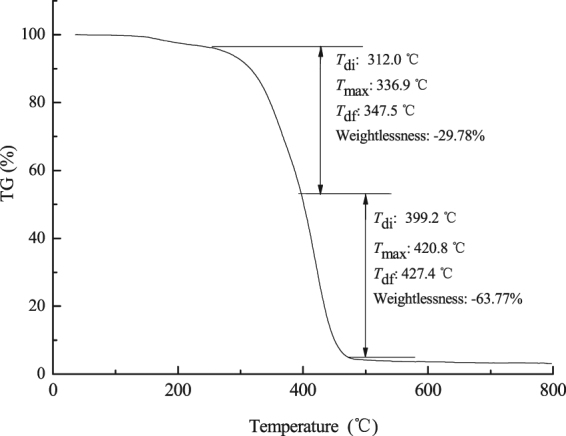
TG curve of the UV-cured product of tri-allyl maleopimarate.

Figure 8 shows the weight loss of the UV-cured product of tri-allyl maleopimarate, recorded while heating the sample from 30 to 800 °C (10 °C /min) in N_2_ atmosphere. A bimodal weight-loss curve was observed with an initial loss at 312.0 °C. More detailed TG data of the UV-cured product is reported in Table 5.

**Table 5:** TG data of UV-cured products of different monomers.

TG	*T*_di_(°C)	*T*_max_(°C)	*T*_df_(°C)	WL(%)
First section thermal decomposition	312.0	336.9	347.5	29.78
Second section thermal decomposition	435.7	474.9	5029	63.77

**Figure 9:**
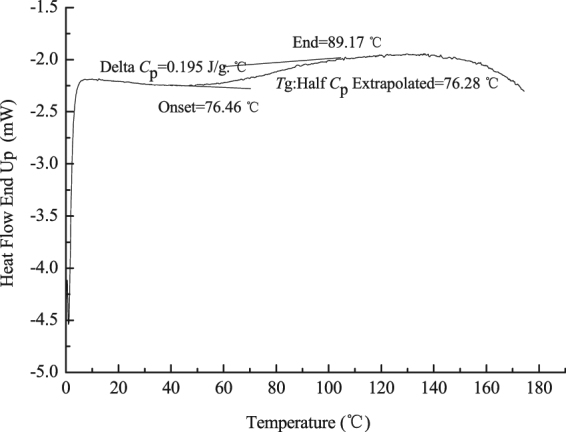
DSC curve of the UV-cured product of tri-allyl maleopimarate.

Figure 9 shows the DSC curve of the cured product of tri-allyl maleopimarate. The following characteristic temperatures can be obserbed: an onset temperature of 76.46 °C, an end temperature of 89.17 °C, and a mid-point of specific heat change (*C*_p_) of 0.195 J/(g·°C). The glass transition temperature of the UV-cured product from tri-allyl maleopimarate was higher than that of allyl maleopimarate (see Figure S9 of the Supplementary Materials). The results showed that the three-dimensional net structure of the polymer formed from tri-allyl maleopimarate had higher thermal stability than the two dimensional cross-linked structure of the polymer formed from allyl maleopimarate.

#### Mechanical properties of the UV-cured polymers of tri-allyl maleopimarate

Table 6 gives the mechanical properties of UV-cured product. The product was a smooth and transparent polymer film. TG data of the UV-cured product derived from the allyl maleopimarate based film is shown in Figure S8 and Table S3 of the Supplementary Materials.

**Table 6:** Mechanical properties of the UV-cured product of tri-allyl maleopimarate.

Properties	Appearance	Adhesion	Pencil hardness	Impact strength	Flexibility	Chemical and water resistance
UV-cured product of tri-allyl maleopimarate	Smooth, transparent	0 grade	2H	>50 cm	>Mandrel 7	Pass

The UV-cured product showed excellent flexibility. An adhesion grade of 0 and an impact strength of over 50 cm were obtained. Due to the introduction of the rosin chain, the cured product passed the acid, alkali, salt, and water resistance tests. A difference between the two UV-cured products lay in their pencil hardness, which was measured to be 2H and 5H for the products of tri-allyl maleopimarate and allyl maleopimarate, respectively (See Table S4). The higher hardness value of the UV-cured product of allyl maleopimarate is attributable to the hardness of the acid anhydride thermally cured product.

#### UV curing mechanism of tri-allyl maleopimarate

**Figure 10:**
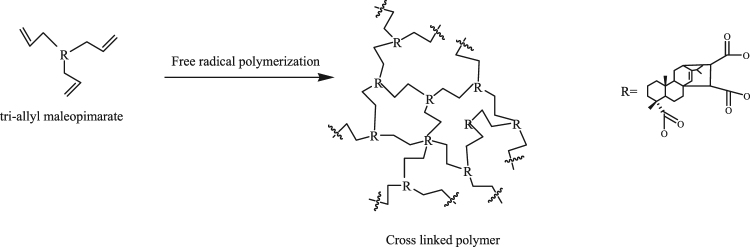
Polymerization mechanism of tri-allyl maleopimarate.

A polymerization mechanism of tri-allyl maleopimarate was proposed and is shown in Fig. 10. The monomer took part in a free-radical polymerization reaction. Tri-allyl maleopimarate, bearing three allyl double bonds, was suitably predisposed for more complex polymerization involving cross-linking; on the other hand, the allyl maleopimarate could only form a polymer with a two dimensional cross-linked structure (see Figure S10).

## Material and Methods

### Materials and equipment

The raw materials used were maleopimaric anhydride (MPA) (GC content 98.4%), allyl chloride, hydroquinone, hexadecyl trimethyl ammonium bromide, sodium hydroxide, N, N-dimethyl formam (DMF), *n*-hexane, benzoin dimethyl ether (photoinitiator 6512), tetrahydrofuran. All reagents were analytically pure and bought from Nanjing Chemical Reagent Co., Ltd., China.

The UV-curing instrument used was an Intelli-ray 600. Intelli-ray 600 utilizes a metal halide type arc-lamp and the radiation flux is 315–400 nm. Analyses were carried out using a GC–2014 gas chromatograph (Shanghai Precision Science Instrument Co Ltd, China), Nicolet 6700 Fourier transform infrared spectrometer (Thermo Nicolet Corporation, USA), 6890 N/5973 N gas chromatograph–mass spectrometer (Agilent, USA), Bruker DRX500 MHz nuclear magnetic resonance, PE–2400 elemental analyzer (PerkinElmer, USA), NDJ–79 rotary viscosimeter (Tongji University, China), Waters 1515 gel permeation chromatography (Waters, USA), and Diamond DSC differential scanning calorimeter (Perkin–Elmer, USA).

### Preparation of sodium maleopimarate

Sodium maleopimarate was synthesized using pimaric acid and NaOH as raw materials. 32.8 g of NaOH, which were dissolved in 100 mL water, were added dropwise to 100 g of pimaric acid. The reaction mixture was stirred at room temperature for 1 h and then subjected to vacuum distillation to obtain sodium maleopimarate. The product was dried at 40 °C and was used as starting material for the following reaction.

### Synthesis of tri-allyl maleopimarate

**Figure 11:**
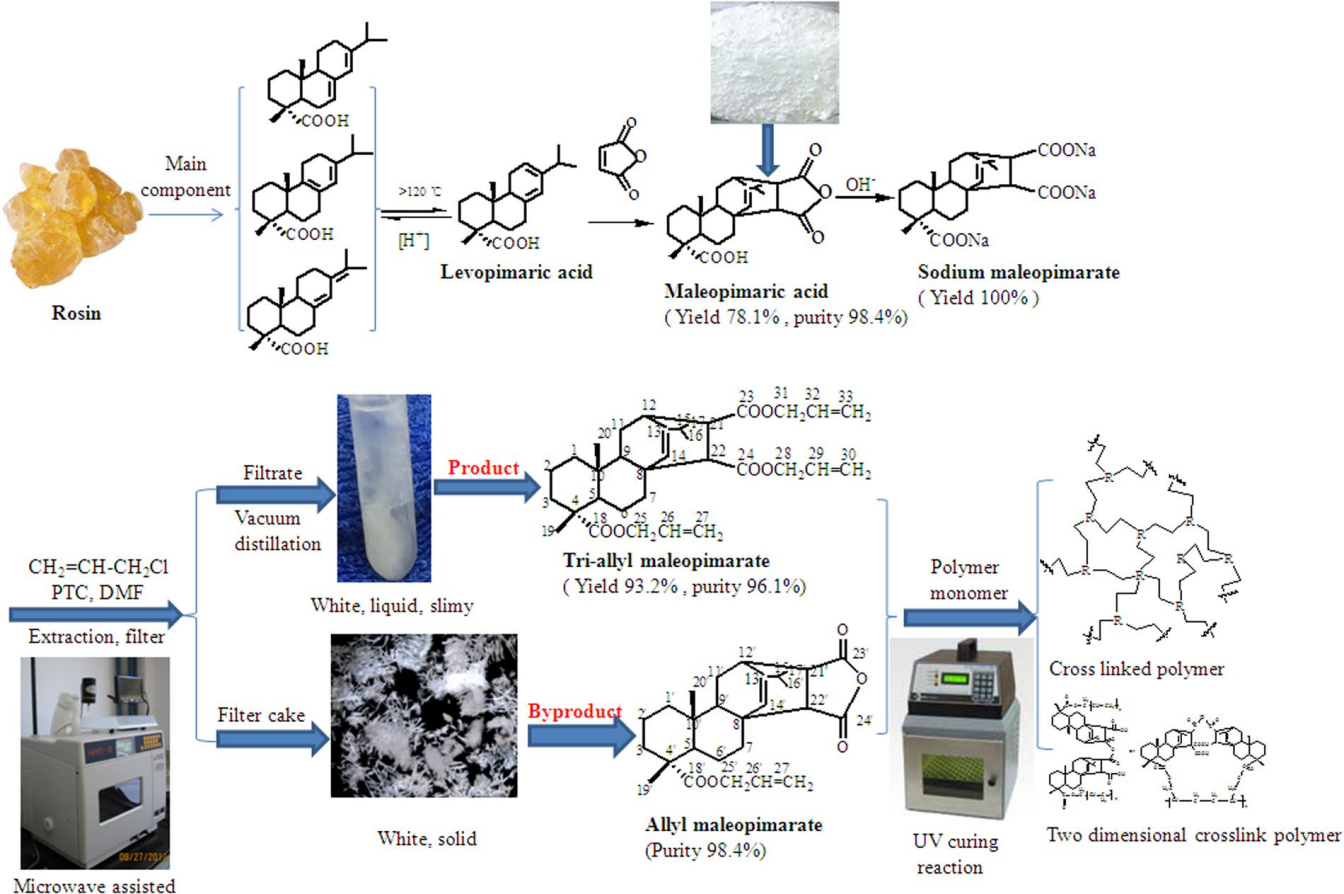
Synthesis of tri-allyl maleopimarate.

Tri-allyl maleopimarate was synthesized from sodium maleopimarate with allyl chloride using DMF as solvent under microwave irradiation (MAS-II microwave workstation, Shanghai Sineo Microwave Chemical Technology Co. Ltd.). The esterification pathway for tri-allyl maleopimarate is outlined in Fig. 11.

Tri-allyl maleopimarate was synthesized using allyl chloride and sodium maleopimarate as materials with *p*-benzoquinone as inhibitor and hexadecyl trimethyl ammonium bromide as phase-transfer catalyst (PTC). The flask was heated with microwave irradiation at 300–600 W to 40–60 °C for 1–3.5 h. The filtrate was extracted three times with *n*-hexane. The extracts were combined and dried using Na_2_SO_4_ for 12 h. The byproduct of allyl maleopimarate was obtained from the filter cake and tri-allyl maleopimarate was obtained from the filtrate after vacuum distillation. The allyl maleopimarate is a white solid, and tri-allyl maleopimarate is a white viscous liquid.

### UV-curing reaction

Tri-allyl maleopimarate was used as the monomer, mixed with the photoinitiator in a fixed proportion using tetrahydrofuran as diluent, and stirred evenly to form a solution. The mixture was then applied onto a tinplate panel (120 × 50 × 2.8 mm), and the thickness of sample was 50 μm. The UV curing reaction was carried out in the following conditions: wave band 315–400 nm, intensity 100%, distance 4.5 cm and time 30 min, then cut off to determine the thermal stability of the UV product. The time required to complete surface drying was then recorded. The same procedure was used for curing allyl maleopimarate.

### Measurements

#### FTIR analysis

FTIR spectra were measured using a Nicolet 6700 FTIR spectrometer over the wavelength range 4000–400 cm^–1^. The UV curing products were smeared onto a KBr crystal plate, then the solvent was allowed to evaporate completely at room temperature.

#### GC and GC–MS analysis

GC (RTX–5 column, 30 m × 0.25 mm × 0.25 μm) was used to analyze tri-allyl maleopimarate at a starting temperature of 240 °C, heating rate of 5 °C/min and final temperature of 270 °C. The sample was held at the final temperature for 40 min. GC-MS (Agilent 6890 N/5973 N, HP–5 column, 30 m × 0.25 mm × 0.25 μm) was conducted to analyze the tri-allyl maleopimarate product.

#### Nuclear magnetic resonance spectroscopy analysis

Nuclear magnetic resonance spectra were recorded on a Bruker (Germany) 500 MHz spectrometer. Deuterated chloroform (CDCl_3_) was used to dissolve samples. ^1^H and ^13^C NMR spectra were obtained using tetramethylsilane (TMS) as an internal standard.

#### Surface drying time

Surface drying time was tested according to the China National Standard: GB 1728–1979(1989) (B method—finger contact method). The drying time was recorded as the period from the moment in which the sample was exposed to the UV irradiation to completion of the surface curing reaction.

#### TG analysis

Thermogravimetric analysis was performed on Perkin-Elmer Diamond TG/DTA with 20 mL/min high-purity nitrogen as purge gas, and the scan rate of the gas was 10 °C/min from 0 to 800 °C.

#### DSC analysis

Differential scanning calorimetry analysis was performed with 20 mL/min high purity nitrogen as purge gas at a scan rate 20 °C/min from −50 to 180 °C.

#### Performance measurement of curing film

The physical properties of the UV cured product from tri-allyl maleopimarate were tested according to the China National Standards GB 9286–1998, GB/T 6739–1996, GB/T 1732–1993, GB/T 1732–1993, and GB/T 1763–1989, respectively. The acid value was determined according to the China National Standard: Test methods for rosin (GB/T 8146–2003). The UV-cured films were stored in a dust-free cabinet for testing purposes.

## Conclusions

This work described a novel method for the synthesis of tri-allyl maleopimarate. The tri-allyl maleopimarate bearing three active vinyl groups were prepared by microwave irradiation and a phase-transfer catalytic reaction. The newly synthesized monomer, consisting of three functional C=C bonds, was found to have plenty of reactive functionalities during free radical polymerization. GC, GC-MS, FTIR, MS, ^1^H NMR, ^13^C NMR and elemental analysis were employed to characterize the physical–chemical properties of tri-allyl maleopimarate, demonstrating that vinyl groups were successfully cited into the structure. Three-dimensional cross-linked polymers were polymerized from tri-allyl maleopimarate and the possible polymerization mechanism of tri-allyl maleopimarate were explored. The introduction of the rosin structure into polymer films can improve the adhesion and mechanical properties, especially in intensity and ductility. The polymers of tri-allyl maleopimarate possess good chemical stability and have a great potential for the coating applications.

## Electronic supplementary material

Supplementary Information
