# Supplementary material for: Synthesis of a multifunctional hard monomer from rosin: the relationship of allyl structure in maleopimarate and UV-curing property
Source: Sci Rep. 2018 Feb 5;8:2399. doi: 10.1038/s41598-018-20695-5 (PMC5799196; doi:10.1038/s41598-018-20695-5)
Supplement: Supplementary file 1 — Supplementary Information [file 41598_2018_20695_MOESM1_ESM.doc]

**Synthesis of a multifunctional hard monomer from rosin: the relationship of allyl structure in maleopimarate and UV-curing property**

Yanju LU^a,b^, Zhendong ZHAO*^a,b^, Liangwu BI^a,b^, Yuxiang CHEN^a,b^, Jing WANG^a^, Shichao XU^a^

a. Institute of Chemical Industry of Forest Products, CAF; National Engineering Laboratory for Biomass Chemical Utilization; Key and Open Laboratory on Forest Chemical Engineering, SFA; Key Laboratory of Biomass Energy and Material, Jiangsu Province, Nanjing 210042, China.

b. Research Institute of Forestry New Technology, CAF, Beijing 100091, China

*Corresponding author: Zhendong Zhao, No.16, Suojin Wucun, Nanjing 210042, P. R. China, Email Address: [zdzhao@189.cn](mailto:zdzhao@189.cn)

**Supplementary information**

Figures S1 (a) and (b) are the FTIR spectra of maleopimaric acid anhydride and sodium maleopimarate, respectively. A detailed analysis of these figures is reported in the manuscript. Figure S1 (c) is the FTIR spectrum of allyl maleopimarate (byproduct of tri-allyl maleopimarate). The main absorption peaks of allyl maleopimarate are as follows: 2932, 2866, 1838, 1769, 1711, 1462 and 1447 cm^-1^. The peaks at 1838 and 1769 cm^-1^ can be assigned to the stretching coupling vibration of C=O from acid anhydride. We can see that the anhydride acid disappeared from sodium maleopimarate (see Figure S1 (b)), but was regenerated in the allyl maleopimarate compound. The peak of C=O at 1689 cm^-1^ disappeared and was converted into the peak of ester the group at 1711 cm^-1^. The peak at 3080 cm^-1^ appeared due to the absorption of the =C-H double bond.

Figure S1 FTIR spectra of allyl maleopimarate and raw material, a. maleopimaric acid anhydride; b. sodium maleopimarate; c. allyl maleopimarate

Figure 2S is the GC trace of allyl maleopimarate, the byproduct of tri-allyl maleopimarate. Its GC content is 98.4% at 26.7 min.

Figure S2 Gas chromatogram of allyl maleopimarate

Typical fragmentation patterns of the byproduct allyl maleopimarate are shown schematically in Figures S3 and [S4](http://onlinelibrary.wiley.com/doi/10.1021/bp000058h/full#fig4). The fragment ion with molecular weight 440 represents the molecular ion [M]^+^. Loss of an ester group from the allyl maleopimarate substituent yielded the fragment [M-OCH_2_CHCH_2_]^+^. The fragment ion with molecular weight 342 is the product of the reverse Diels–Alder reaction. The base peak is the fragment ion with molecular weight 197, which randomly generates small fragment ions.


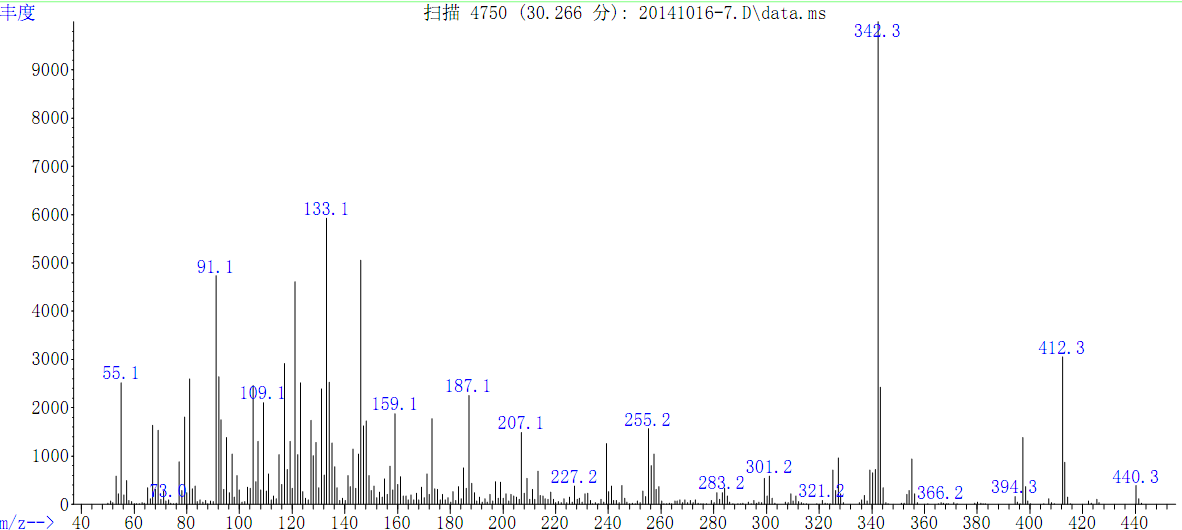


Figure S3 Mass spectrum of allyl maleopimarate

Figure S4 Fragment ions of allyl maleopimarate

**Hydrogen nuclear magnetic resonance spectroscopy analysis of allyl maleopimarate**

The chemical structure of allyl maleopimarate was confirmed by hydrogen nuclear magnetic resonance spectroscopy (Figure S5) using a Bruker 500 MHz spectrometer at room temperature with CDCl_3_ as both solvent and internal reference.

The hydrogen nuclear magnetic resonance spectroscopy spectrum of allyl maleopimarate was shown in Figure S5. The carbon atom number of allyl maleopimarate was shown in Eq. (1). The characteristic peaks of allyl maleopimarate are as follows: *δ* 5.58~5.97 are the multiple peaks of C26—H, *δ* 5.40 and 5.39 are the double peaks of C27—H, *δ* 5.30 and 5.27 are the double peaks of C27—H, *δ* 5.34 is the single peak of C14—H, *δ* 4.63 and 4.62 are the double peaks of C25—H. The results confirm that the vinyl double bond was successfully added to the product. In addition, the double peaks of C21—H at *δ* 3.16 and 3.13 and the single peak of C22—H at *δ* 3.12 show that the anhydride is also present in the compound.


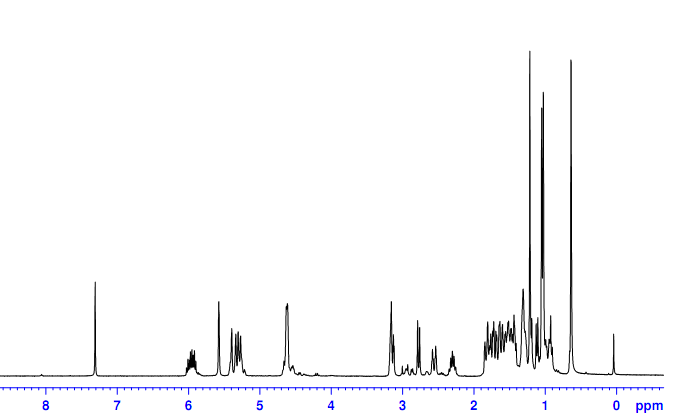


Figure S5 Hydrogen nuclear magnetic resonance spectroscopy spectrum of allyl maleopimarate

Other peaks of allyl maleopimarate were as follows: *δ*: 1.28~1.19 (m, 2H, C1—H, C2—H), 1.47~1.44 (m, 2H, C1—H, C2—H), 1.81~1.84 (t, 1H, C3—H), 1.69~1.63 (1H, C3—H), 2.30 (1H, C5—H), 1.59~1.51 (m, 1H, C6—H, C7—H, C11—H), 1.28~1.19 (m, 1H, C6—H, C7—H, C11—H), 1.41 (1H, C9—H), 1.79~2.58 (t, 1H, C12—H), 5.34 (s, 1H, C14—H), 2.53 (1H, C15—H), 0.99 and 0.98 (3H, C16—H), 0.94 and 0.92 (3H, C17—H), 1.31 (s, 3H, C19—H), 1.13 (s, 3H, C20—H), 3.16 and 3.13 (d, 1H, C21—H), 3.12 (s, 1H, C22—H), 4.63 and 4.62 (d, 2H, C25—H), 5.97~5.58 (m, H, C26—H), 5.40 (d, H, C27—H), 5.30 and 5.27 (d, H, C27—H).

**Carbon nuclear magnetic resonance spectroscopy analysis of allyl maleopimarate**

The carbon nuclear magnetic resonance spectroscopy chemical shifts and the carbon nuclear magnetic resonance spectroscopy signals of allyl maleopimarate were shown in Figure S6 and Table S1. The signals at *δ*=77.25, 77.00, and 76.75 ppm are due to the presence of chloroform, used as an internal reference. The signals of the C=O bonds, C-18’, C-23’ and C-24’, appear at *δ*=177.7, 172.2, and 170.5 ppm, respectively. The signals of the allyl double bonds appear at *δ*=131.8 (C-26’), 117.5 (C-27’), 147.6(C-13’) and 124.7(C-14’) ppm.


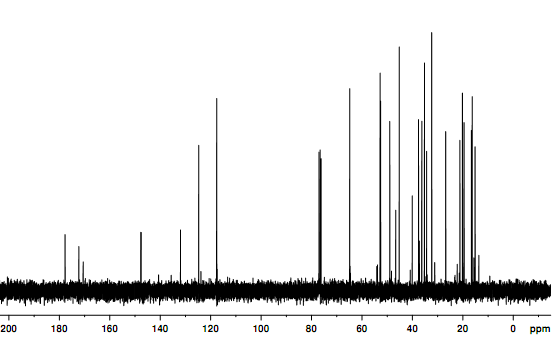


Figure S6 Carbon nuclear magnetic resonance spectroscopy spectrum of allyl maleopimarate

Table S1 Carbon nuclear magnetic resonance spectroscopy chemical shift of each carbon atom in allyl maleopimarate

| Carbon number | *δ* | Carbon number | *δ* |
| --- | --- | --- | --- |
| 1’ | 45.2 | 15’ | 34.3 |
| 2’ | 19.5 | 16’ | 32.2 |
| 3’ | 39.9 | 17’ | 31.1 |
| 4’ | 48.9 | 18’ | 177.7 |
| 5’ | 53.8 | 19’ | 22.2 |
| 6’ | 20.1 | 20’ | 26.7 |
| 7’ | 35.2 | 21’ | 37.2 |
| 8’ | 16.5 | 22’ | 54.1 |
| 9’ | 52.7 | 23’ | 172.2 |
| 10’ | 37.5 | 24’ | 170.5 |
| 11’ | 32.3 | 25’ | 64.7 |
| 12’ | 19.9 | 26’ | 131.9 |
| 13’ | 147.6 | 27’ | 117.6 |
| 14’ | 124.7 |  |  |

**Elemental analysis**

The elemental analysis data of allyl maleopimarate, expressed in percentages, are as follows: *ω*(C) =74.3(73.6), *ω*(H)=8.3(8.2), *ω*(O)=19.0(18.2). Values inside the parentheses indicate the theoretical data.

**Physical properties of allyl maleopimarate**

The byproduct allyl maleopimarate was obtained as a white solid with a melting point 141.8~142.3°C.

**UV-polymerization performance of allyl maleopimarate**

The UV-polymerization reaction conditions were as follows: illumination distance 4.5 cm, illumination intensity 100%, photo initiator 6512.

**FTIR monitoring of allyl maleopimarate conversion**

The FTIR spectra of the polymerized product from allyl maleopimarate before and after polymerization under UV irradiation are shown in Figure S7. Before polymerization, it can be seen that there were characteristic absorption peaks at 1683 and 1770 cm^-1^ due to anhydride, and an absorption peak at 1647 cm^-1^ due to the C=C double bonds. After 30 min of polymerization, the C=C double bonds were gradually polymerized, and the corresponding peak area (at 1647 cm^-1^) decreased. At the same time, the absorption peaks at 1683 and 1770 cm^-1^ disappeared. These results indicate that the UV-polymerization reaction of allyl maleopimarate not only opens the double bonds of the free radical, but also favors thermal polymerization, which opens the anhydride ring (see Figure S10).

Figure S7 FTIR spectra of allyl maleopimarate before and after UV-polymerization, a. 0 min; b. 30 min

**Effects of the operating conditions on the surface drying time from allyl maleopimarate**

The surface drying time of allyl maleopimarate was of just a few seconds and decreased with increasing photoinitiator dosages up to 6%.

Table S2 Surface drying time from allyl maleopimarate under different polymerization conditions

| Polymerization conditions | | Surface drying time(s) |
| --- | --- | --- |
| Photo-initiator dosage (%) | 1 | 24 |
|  | 2 | 15 |
|  | 3 | 14 |
|  | 4 | 12 |
|  | 5 | 11 |
|  | 6 | 10 |
|  | 7 | 9 |
|  | 8 | 9 |
|  | 9 | 9 |
|  | 10 | 9 |
| Illumination distance (cm) | 4.5 | 15 |
|  | 9.0 | 20 |
|  | 13.5 | 30 |
|  | 18.0 | 36 |
|  | 22.5 | 40 |
| Illumination intensity (%) | 50 | 30 |
|  | 60 | 28 |
|  | 70 | 24 |
|  | 80 | 22 |
|  | 90 | 18 |
|  | 100 | 15 |

The thermal polymerization of the anhydride led to the higher initial weight loss temperature, which was found to be 323.6°C for the polymer from allyl maleopimarate. More detailed TG data of the UV-cured product from allyl maleopimarate are reported in Table S3.

Figure S8 TG curve of UV-cured product from allyl maleopimarate

Table S3 Mechanical properties of UV-cured product from allyl maleopimarate

|  | TG | *T*_di_(^o^C) | *T*_max_(^o^C) | *T*_df_(^o^C) | WL(%) |
| --- | --- | --- | --- | --- | --- |
| First section thermal decomposition | | 312.0 | 336.9 | 347.5 | 29.78 |
| Second section thermal decomposition | | 323.6 | 335.2 | 346.8 | 39.43 |

Differential scanning calorimetry was performed with 20 mL/min high-purity nitrogen as purge gas. A two-stage heating program was adopted; the heating and cooling rates were always of 20°C/min. During the first heating stage, the temperature was held at 0°C for 1 min, then raised to 150°C and held for 2.5 min. The sample was then cooled back down to 0°C and held at that temperature for 1 min. The second heating stage consisted in heating the sample from 0 to 150°C. Data recorded during the second heating process provide the *T*g value. The UV-cured product obtained from allyl maleopimarate showed an onset temperature of 52.99°C, an end temperature of 57.76°C, the range of thermal change is from 52.99 to 57.76°C, with mid-point of specific heat change (*C*_p_) of 0.132 J/(g·°C). The glass transition temperature is therefore 56.52°C. The result is consistent with the definition of the glass transition temperature as the one in correspondence of which a change in heat flow is observed to the phase change.

Figure S9 DSC curves of UV cured product from allyl maleopimarate, a. first heating process; b. second heating process

Table S4 Mechanical properties of different UV-cured products from allyl maleopimarate

| Properties | Appearance | Adhesion | Pencil hardness | Impact strength | Flexibility | Chemical and water resistance |
| --- | --- | --- | --- | --- | --- | --- |
| UV-cured product of allyl maleopimarate | Smooth, transparent | 0 grade | 5H | >50 cm | >  Mandrel 7 | Pass |

Allyl maleopimarate could only form a polymer with a two dimensional cross-linked structure.

Figure S10 Polymerization mechanism of allyl maleopimarate
